# Correlation analysis of HER2 expression with clinicopathological features and prognosis based on data from 444 patients with urothelial carcinoma

**DOI:** 10.1186/s12894-026-02088-3

**Published:** 2026-02-24

**Authors:** Jingyi Luo, Ziyi Cheng, Lihang Chen, Xiaorui Li, Zhixun Guo, Jinfeng Wu, Liefu Ye, Haijian Huang, Jiawen Wang, Yongbao Wei

**Affiliations:** 1 Fujian Medical University, Fuzhou, China; 2 Shengli Clinical Medical College of Fujian Medical University, Fujian Medical University, Fuzhou, China; 3 Department of Urology, Fuzhou University Affiliated Provincial Hospital, Fuzhou, 350001 China; 4 Department of Pathology, Fuzhou University Affiliated Provincial Hospital, Fuzhou, 350001 China

**Keywords:** Human epidermal growth factor receptor 2, Prognosis, Urothelial carcinoma

## Abstract

**Background:**

Urothelial carcinoma (UC) is a common malignancy with a poor prognosis in advanced stages, characterized by high heterogeneity, recurrence risk, and chemotherapy resistance. There is a clinical lack of reliable prognostic markers. Human epidermal growth factor receptor 2 (HER2) is a key oncogene and therapeutic target. Its correlation with clinicopathological features in Chinese UC patients remains controversial, leading to unclear biomarker value and limiting the precise application of therapies like RC48-ADC. Therefore, clarifying the association between HER2 expression and UC pathological staging and prognosis is crucial for optimizing treatment.

**Methods:**

Clinical data of 444 UC patients admitted to Fujian Provincial Hospital from January 2020 to August 2024 were retrospectively analyzed. HER2 expression was detected by immunohistochemistry (IHC) and fluorescence in situ hybridization (FISH). Spearman’s rank correlation coefficient was used to clarify the association between HER2 and clinicopathological characteristics, and Kaplan-Meier method and Cox regression model were applied to analyze its prognostic value.

**Results:**

The high HER2 expression rate (IHC 2+/3+) was 40.05%. High HER2 expression was significantly correlated with Age, History, Routine Urine Test indices, Pathological Grade, Ki67 index, p53 expression, and treatment options (all *P*<0.05). Multivariate Cox regression analysis confirmed that high HER2 expression was an independent risk factor for UC Recurrence (HR=2.496, *P*=0.044) but not significantly associated with Metastasis (*P*=0.923).

**Conclusion:**

HER2 is highly expressed in UC patients in the Fujian region, correlating with aggressive clinicopathological features. It can serve as a reliable biomarker for predicting recurrence risk, guiding postoperative management and targeted therapy selection.

**Supplementary Information:**

The online version contains supplementary material available at 10.1186/s12894-026-02088-3.

## Introduction

Urothelial carcinoma(UC) is one of the most common malignant tumors of the urinary system [1]. Notably, advanced UC patients have a poor prognosis and short survival time [2]. Behind this grim situation lies UC’s high heterogeneity, high recurrence risk, and chemoresistance. These factors collectively lead to the clinical dilemma of lacking reliable prognostic indicators.

Human epidermal growth factor receptor 2(HER2) is a key proto-oncogene that regulates cell proliferation, invasion, and metastasis. Its abnormal expression is closely associated with adverse prognoses in breast cancer and gastric cancer [3, 4], and it has become an important marker for targeted therapy. Recent studies indicate that HER2 is frequently expressed in Chinese UC patients; however, its association with clinicopathological features remains inconsistent, limiting its clinical utility as a prognostic biomarker. One research results indicate a statistically significant difference in the distribution of HER2 positivity according to the pathological grades of UC [5]. In contrast, another study reported no significant association between HER2 overexpression and tumor staging or lymph node status [6]. This cognitive limitation greatly hinders the exploration of the value of HER2-targeted therapy in the precise treatment of UC. These conflicting conclusions make it unclear whether HER2 can serve as a reliable biomarker, preventing clinicians from effectively selecting patients most likely to benefit from HER2-targeted therapy and thus severely restricting its application in precision medicine. Especially with the gradual application of anti-HER2 antibody-drug conjugates represented by RC48-ADC in UC patients [7], the clinical value of the HER2 target for UC patients has become increasingly prominent. Therefore, clarifying the relationship between HER2 expression and pathological stage as well as prognosis is crucial for identifying potential beneficiaries.

In summary, this study intends to collect relevant clinical data of UC patients in recent years, investigate the expression of HER2, clarify the correlation between HER2 expression and different clinicopathological characteristics of UC patients and further explore the relationship between HER2 and prognosis through survival analysis. Given the controversial prognostic value of HER2 in UC, this study aims to investigate its feasibility as a potential risk stratification marker rather than directly defining it as a definitive independent prognostic factor, to provide a basis for optimizing HER2-targeted therapy.

## Methods

### Patients

This study is a retrospective analysis, including 444 patients with urothelial carcinoma who were pathologically diagnosed at Fujian Provincial Hospital from January 2020 to August 2024. Inclusion criteria: ①Cases from January 2020 to August 2024; ②HER2 immunohistochemistry and/or genetic testing should be conducted before treatment; ③Complete the MRI examination before treatment; ④Possess complete and traceable clinical medical records. Exclusion criteria: ①Combined with other malignant tumors; ②Those lacking key information. The project has been reviewed and approved by the Ethics Committee of Fuzhou University Affiliated Provincial Hospital. Collect the basic information of the patients, such as name, age, gender, previous history of bladder tumor, history of chronic bladder inflammation, comorbidities (including hypertension, diabetes, other or multiple diseases), blood routine (including white blood cell count, neutrophil count, lymphocyte count, hemoglobin quantity, platelet count), biochemical tests (including total protein, albumin, globulin, creatinine, blood calcium, apolipoprotein), urine routine (PH value, white blood cell count, red blood cell count, epithelial cell count), magnetic resonance tumor imaging features (including the maximum diameter of the lesion), pathological data (including lesion morphology, cTNM stage, pathological grade, Ki67, P53, HER2), clinical treatment (including whether immediate perfusion is performed after surgery, maintenance perfusion method, maintenance perfusion course, neoadjuvant therapy), follow-up Visit records (including survival period, recurrence and metastasis conditions), and construct a standardized and dynamically updated clinical database.

### HER2 expression detection

The expression of HER2 protein was detected by immunohistochemistry (IHC), and the amplification of HER2 gene was detected by fluorescence in situ hybridization (FISH). The multi-parameter plain scan and enhanced examination of bladder MRI were used to evaluate the structure, function and lesion conditions of the bladder and surrounding tissues, diagnose bladder diseases, clarify the nature of the lesion and determine the severity of the disease. MRI is performed in the supine position, using 18-channel volumetric coils and 32-channel integrated spinal coils, belonging to the 3.0T MRI system (magnetom Prisma; Siemens Healthcare, Erlangen, Germany). The obtained MR Images include T2-weighted images and diffusion-weighted images, covering three planes: the maximum axial position, the oblique coronal position and the sagittal position [8].

The reference standard is based on the “Clinical Pathological Expert Consensus on the Detection of Human Epidermal Growth Factor Receptor 2 in Urothelial Carcinoma in China” published in the Chinese Journal of Oncology in October 2021. Incomplete and weakly stained membranes of non-staining or < 10% invasive cancer cells correspond to a score of 0; incomplete and weakly stained membranes of ≥ 10% invasive cancer cells correspond to a score of 1+; weakly to moderately intense intact cell membrane staining of ≥ 10% invasive cancer cells corresponds to a score of 2+; and intact fine membranes of ≥ 10% invasive cancer cells correspond to a score of ≥ 10% Strong staining of the cell membrane corresponds to a score of 3+. Low expression is defined as HER2 0 or 1+, and high expression is defined as HER2 2 + or 3+.

### Statistical analysis

All data were statistically analyzed using IBM SPSS Statistic 27.0 statistical software. Using statistical methods HER2 expression is related to age, gender, previous history of bladder tumor, history of chronic bladder inflammation, comorbidities (including hypertension, diabetes, other or multiple diseases), blood routine (including white blood cell count, neutrophil count, lymphocyte count, hemoglobin quantity, platelet count), biochemical tests (including total protein, albumin, globulin, creatinine, blood calcium, apolipoprotein) Urine routine (pH value, white blood cell count, red blood cell count, epithelial cell count), magnetic resonance tumor imaging features (including maximum diameter of the lesion), pathological data (including lesion morphology, cTNM stage, pathological grade, Ki67, p53, HER2), clinical treatment (including whether immediate perfusion is performed after surgery, maintenance perfusion method, maintenance perfusion course, neoadjuvant therapy Analyze the relationship between treatment records and follow-up records (including survival period, recurrence and metastasis conditions). The Spearman correlation analysis method was used to evaluate the association between HER2 expression and age, gender, previous history of bladder tumor, history of chronic bladder inflammation, comorbidities, blood routine, biochemical tests, urine routine, magnetic resonance tumor imaging features, pathological data, and clinical treatment. Kaplan-Meier survival analysis, Cox proportional hazards regression model and other methods were used to evaluate the impact of HER2 expression on the prognosis of patients (including survival period, recurrence and metastasis). Meanwhile, a multivariate analysis was conducted to eliminate the interference of age, gender, blood routine, and biochemical tests, and to determine the independent prognostic value of HER2 expression. A *P* value < 0.05 was statistically significant.

## Results

### Clinical features

**Fig. 1:**
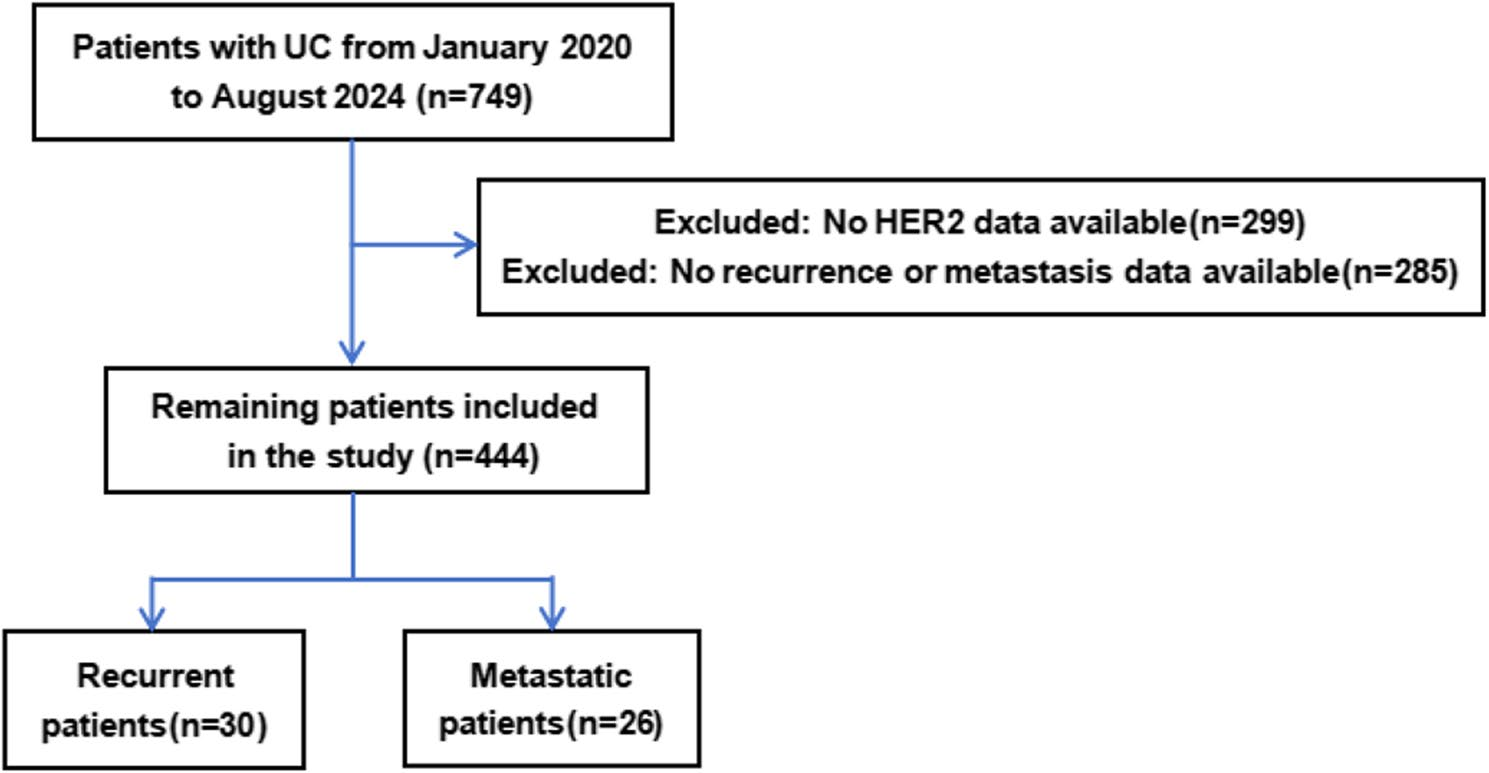
Study Design Flowchart

This study collected case data from 749 patients with UC at the Provincial Hospital Affiliated to Fuzhou University from January 2020 to August 2024. Through inclusion and exclusion criteria, data from 444 patients with valid HER2 and prognosis data were finally included. The flowchart of the study design is shown in Fig. 1.

The basic clinical features of the patients are shown in Table 1. Among the valid data, most patients were males (87.0%); the mean age of the patients was approximately 66 years, with a standard deviation of about 11.026; 87.8% of patients had a maximum tumor diameter≥1 cm; the proportion of patients with high-grade UC was 72.5%, which was significantly higher than that of low-grade UC. Regarding pathological staging, the cT stage was mainly cT1(38.4%) and cT2(33.2%), and among cases with different infiltration depths, UMIUC accounted for 38.4%; in the cN stage, cN0 accounted for the largest proportion (75.8%); and in the cM stage, M0 accounted for the largest proportion (87.5%). The lesion shapes were mainly cauliflower (43.5%) and mass (30.1%). There were 265 patients (59.95%) with low HER2 expression and 177 patients (40.05%) with high HER2 expression. According to medical records as of February 2025, the recurrence rate of these 444 patients was approximately 6.76%, and the metastasis rate was approximately 5.86%.

**Table 1:**
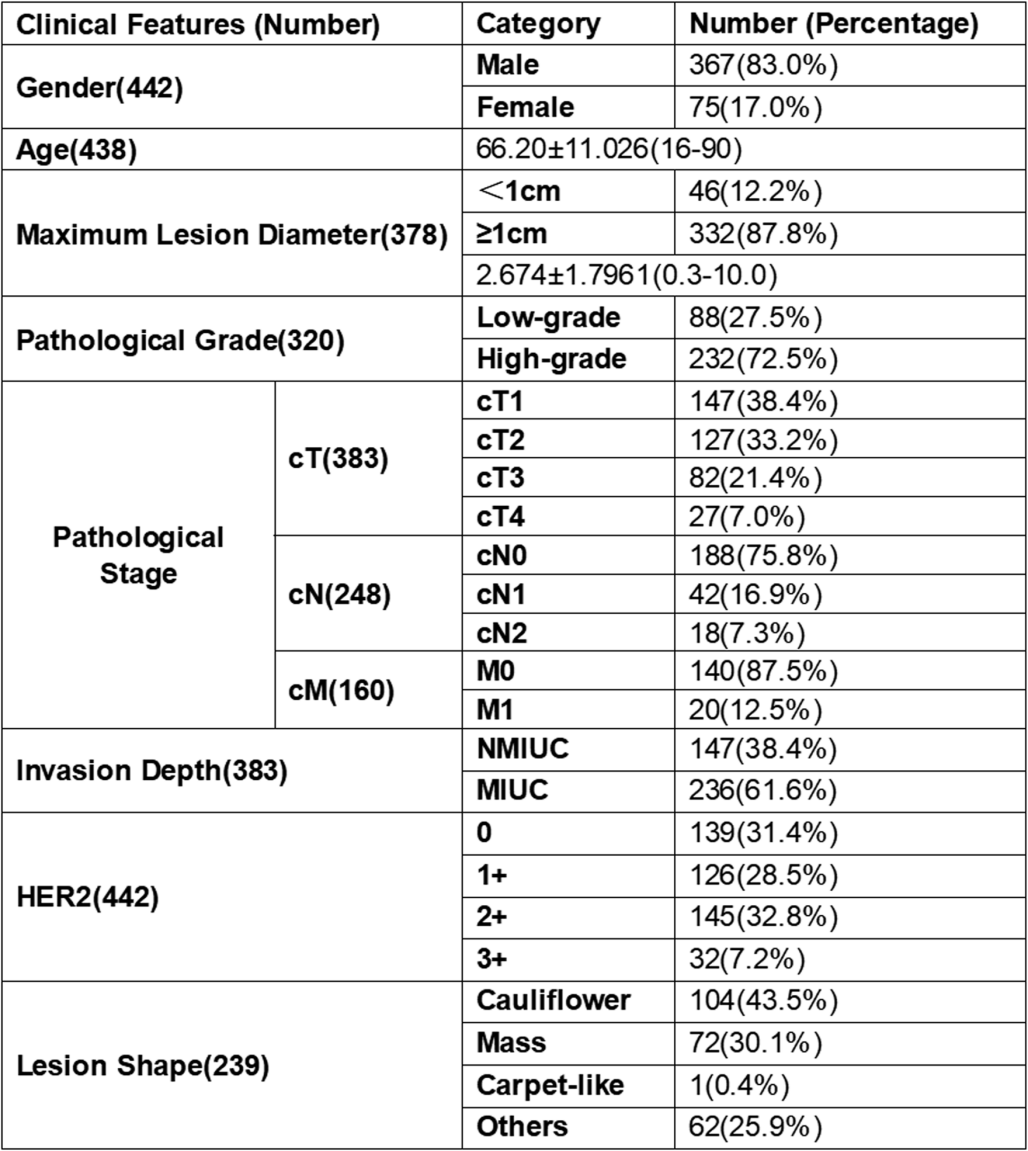
Basic Clinical Features of Patients

### Correlation between clinical features and HER2 in patients with UC

For the valid data of 444 cases in this study, Spearman’s rank correlation analysis was used to calculate the Spearman correlation coefficient between relevant clinical data and HER2, so as to explore the correlation between variables. The specific results are shown in Table 2.

**Table 2:**
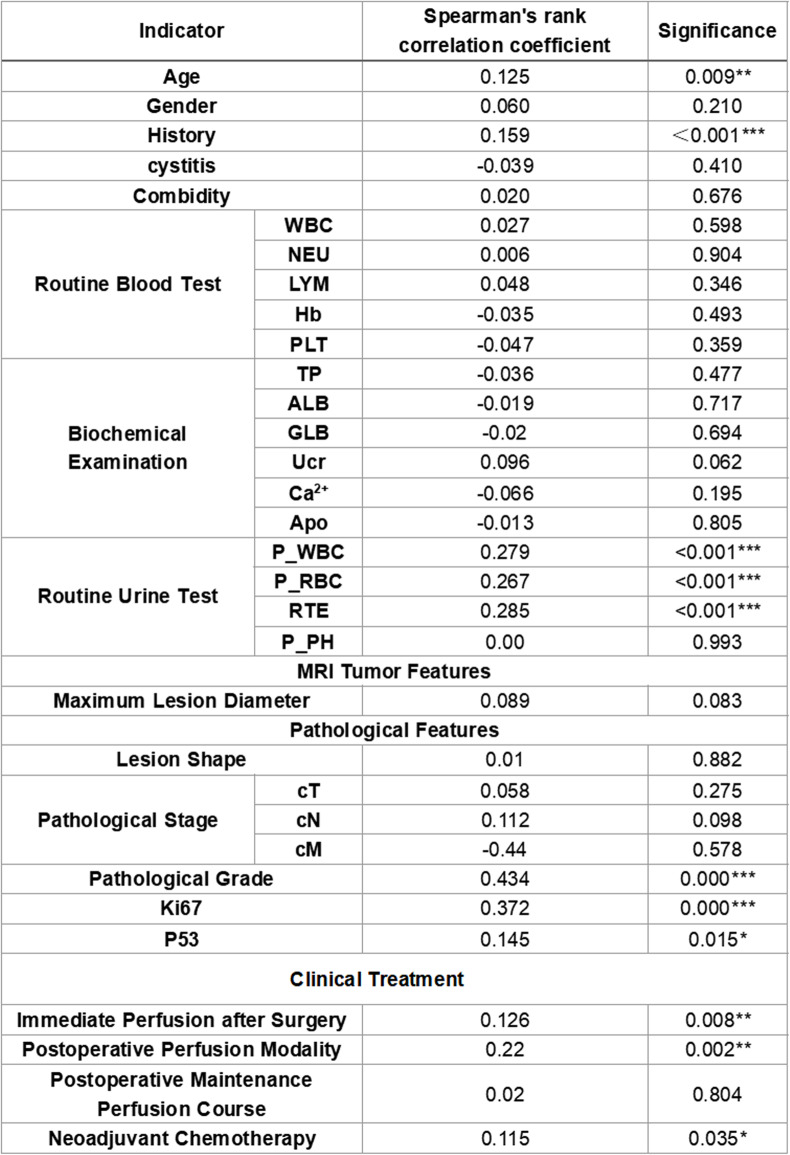
Data on Correlation Analysis between Various Clinical Features of Patients and HER2

Spearman's rank correlation analysis was used.

*p<0.05

**p<0.01

***p<0.001

In the baseline clinical status, the age, history of bladder cancer, and routine urine test of patients with urothelial carcinoma were significantly correlated with high HER2 expression. The Spearman correlation coefficient between HER2 expression and age was 0.125(*p* = 0.009), suggesting that the older the patient, the higher the possibility of high HER2 expression. The Spearman correlation coefficient between HER2 expression and history of bladder cancer was 0.159(*p* < 0.001), suggesting that patients with a history of bladder cancer had a higher possibility of high HER2 expression. The Spearman correlation coefficients between HER2 expression and white blood cells(cells/HP), red blood cells(cells/HP), and epithelial cells(cells/HP) in the routine urine test were 0.279(*p* < 0.001), 0.267(*p* < 0.001), and 0.285(*p* < 0.001), respectively. This suggests that the higher the values of white blood cells(cells/HP), red blood cells(cells/HP), and epithelial cells(cells/HP) in the patient’s routine urine test, the higher the possibility of high HER2 expression.

In the pathological data, the pathological grade, Ki67, and p53 of patients with UC were significantly correlated with high HER2 expression. The Spearman correlation coefficient between HER2 expression and pathological grade was 0.434(*p* = 0.000), suggesting that the higher the patient’s pathological grade, the higher the possibility of high HER2 expression. The Spearman correlation coefficients between HER2 expression and Ki67, p53 were 0.372(*p* = 0.000) and 0.145(*p* = 0.015), respectively, suggesting that the higher the expression of Ki67 and p53 in patients, the higher the possibility of high HER2 expression. The Spearman correlation coefficient between HER2 expression and cN (clinical nodal status) was 0.098. This marginal correlation (0.05 < *P* < 0.10) suggests a potential weak positive association between HER2 expression and tumor size, and this result is hypothesis-generating rather than definitive.

Further analysis of the relationship between HER2 expression and tumor invasion depth showed that although the high HER2 expression rate was numerically higher in patients with muscle-invasive urothelial carcinoma (46.3%) than in superficial cases (35.0%), this difference was not statistically significant (χ²=2.00, *P* = 0.157). This result suggests that high HER2 expression may not be clearly associated with the invasion depth of urothelial carcinoma in our study cohort.

The Kaplan-Meier survival curve was plotted to compare cumulative recurrence-free survival among four subgroups: superficial urothelial carcinoma (UC) with low HER2 expression, superficial UC with high HER2 expression, muscle-invasive UC with low HER2 expression, and muscle-invasive UC with high HER2 expression. The Log-rank test showed no statistically significant difference in cumulative recurrence-free survival across the four subgroups (χ²=4.627, *P* = 0.201). However, visual inspection of the survival curves revealed distinct trends: the muscle-invasive UC with high HER2 expression subgroup exhibited the steepest decline in survival probability, indicating the poorest prognosis, whereas the two superficial UC subgroups (regardless of HER2 expression) displayed relatively flat and overlapping survival curves, suggesting similar favorable prognosis. The muscle-invasive UC with low HER2 expression subgroup showed an intermediate survival trend between the superficial subgroups and the muscle-invasive UC with high HER2 expression subgroup.

Notably, the Spearman correlation coefficient between HER2 expression and the maximum diameter of the lesion was 0.083. This marginal correlation (0.05 < *P* < 0.10) suggests a potential weak positive trend between HER2 expression and tumor size, and this result is hypothesis-generating rather than definitive.

In clinical treatment, whether patients with urothelial carcinoma received immediate postoperative perfusion, the mode of postoperative perfusion, and the choice of neoadjuvant treatment mode were all significantly correlated with HER2. 37.9% of patients with urothelial carcinoma received immediate postoperative perfusion, among which 3.0% chose bacillus Calmette-Guérin (BCG), while 16.8% of patients chose neoadjuvant treatment, among which the number of patients choosing chemotherapy was the largest (accounting for 11.2% of the total). Analysis showed that the Spearman correlation coefficients between HER2 expression and whether to receive immediate postoperative perfusion, the mode of postoperative perfusion, and the choice of neoadjuvant treatment mode were 0.126(*p* = 0.008), 0.22(*p* = 0.002), and 0.115(*p* = 0.035), respectively. This suggests that the expression level of HER2 has a certain correlation with the choice of treatment plan.

### Correlation between prognosis and HER2 in patients with urothelial carcinoma

According to different HER2 expression statuses, the data were divided into two groups: the HER2 low expression group (0, 1+) and the HER2 high expression group (2+, 3+). The Kaplan-Meier method and Cox proportional hazards regression model were used to analyze the postoperative recurrence, metastasis, and HER2 of patients, respectively.

The starting point of Recurrence-Free Survival (RFS) was defined as the date of surgery, and the endpoint as the date of first confirmed diagnosis of tumor recurrence (based on pathological or radiological evidence). If no recurrence was documented at the last follow-up, the last follow-up date was set as the censoring point.

The starting point of Metastasis-Free Survival (MFS) was defined as the date of surgery, and the endpoint as the date of first confirmed diagnosis of distant metastasis (e.g., pulmonary, hepatic, osseous metastasis, etc., based on radiological evidence combined with pathological/needle biopsy evidence). If no distant metastasis was documented at the last follow-up, the last follow-up date was set as the censoring point.

### Correlation between HER2 and recurrence of urothelial carcinoma

Among the 444 patients under follow-up, 30 developed recurrence and 414 were censored, with an overall recurrence rate of 6.76%. The median survival time of the HER2 low-expression group was 9 months (standard error [SE] = 0.574, 95% confidence interval [95% CI]: 7.876–10.124), while that of the high-expression group was 8 months (SE = 0.959, 95% CI: 6.120–9.880).

**Fig. 2:**
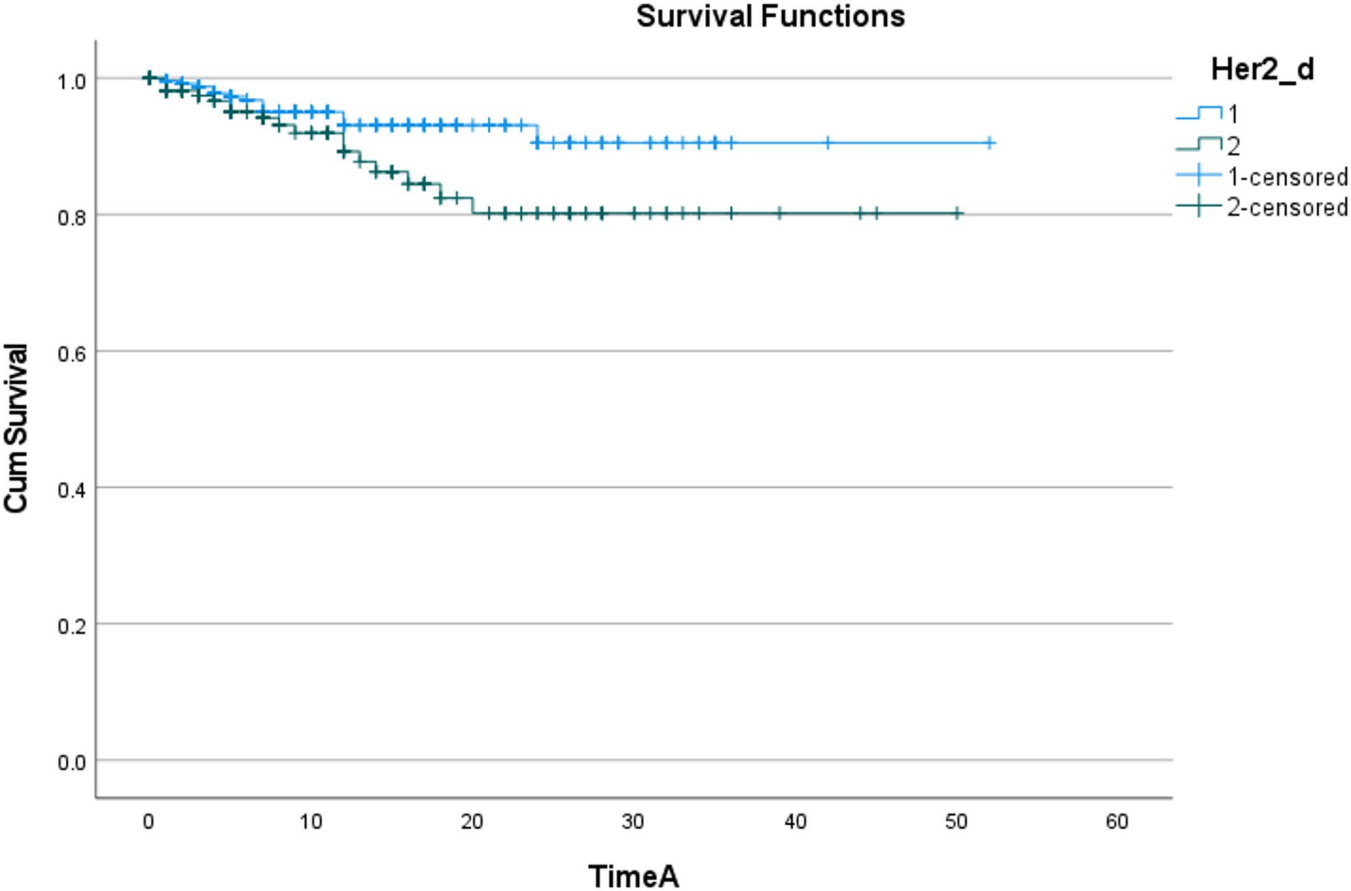
Recurrence Survival Curves of Patients with Different HER2 Expression Statuses

The Kaplan-Meier method was used to draw the recurrence-free survival curve of patients with different HER2 expression statuses (grouped by Her2_d) (as shown in Fig. 2), and the Log-Rank test was used to compare the difference in recurrence risk between groups. The results showed that there was a marginal statistical difference in the cumulative recurrence risk between the two groups(*P* = 0.052), and the survival curve showed a clear separation trend. This indicates that HER2 has a potential impact on recurrence, and different levels of Her2_d have a significant distinguishing effect on survival outcomes, whose potential impact is worthy of further exploration.

**Fig. 3:**
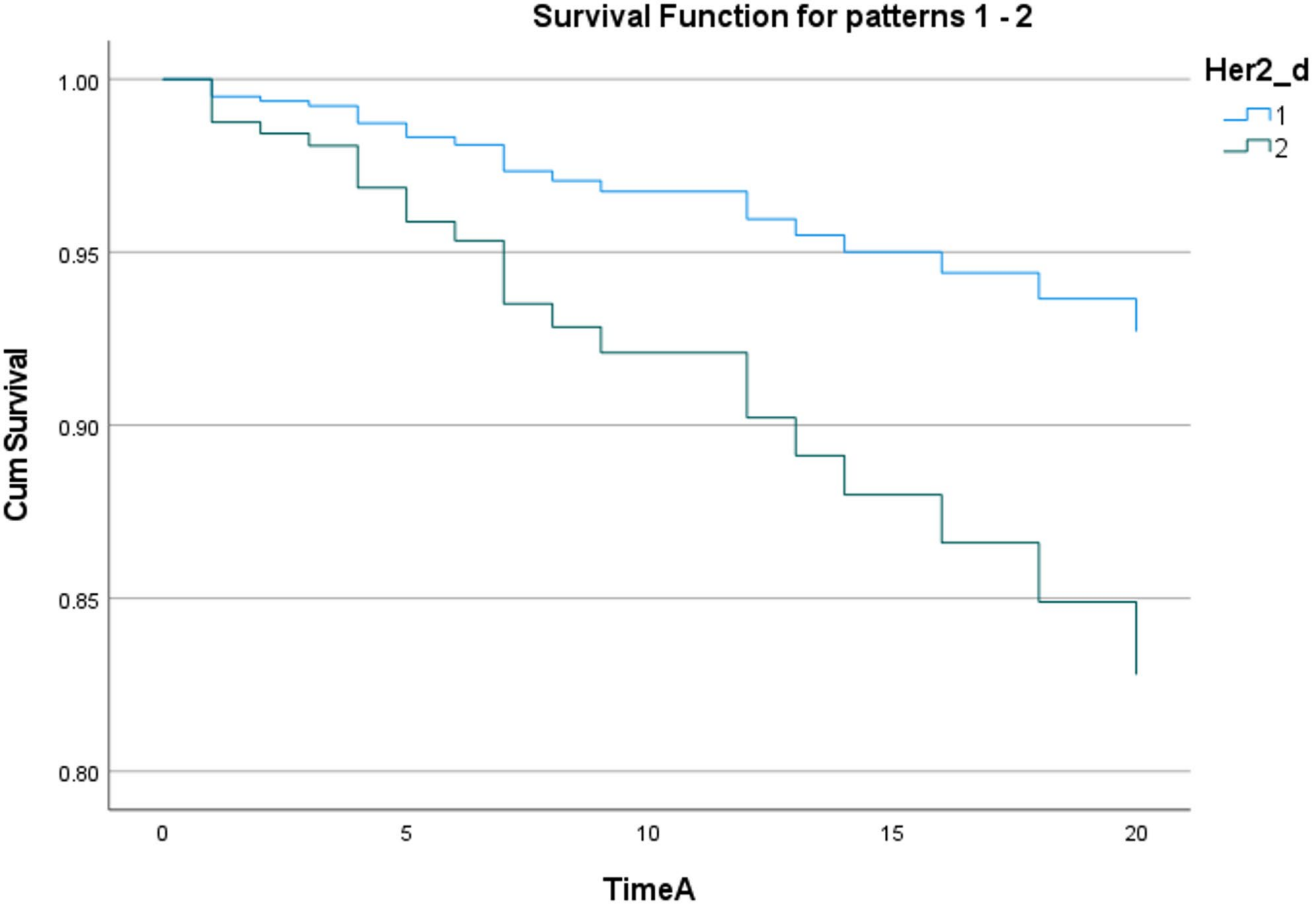
Recurrence Cox Proportional Hazards Regression Models of Patients with Different HER2 Expression Statuses

A multivariate Cox proportional hazards regression model was further constructed, including Her2_d, Age, Gender, and a number of routine blood test and biochemical indicators, to analyze the independent impact of each factor on the recurrence of urothelial carcinoma (as shown in Fig. 3). The Omnibus test of model coefficients showed that after including the variables, the model had statistical significance in predicting recurrence risk (overall chi-square = 43.578, df = 14, *P* < 0.001). After adjusting for age, gender, and other hematological indicators, Her2_d was an independent risk factor for the recurrence of urothelial carcinoma (Wald χ²=4.058, df = 1, *P* = 0.044; HR = 2.496). That is, when Her2_d was grouped as high expression, the recurrence risk of patients was 2.496 times that of the low expression group.

### Correlation between HER2 and metastasis of urothelial carcinoma

Among the 444 followed-up patients, 26 developed metastasis and 418 were censored, with a metastasis rate of 5.86%. The median survival time of the HER2 low-expression group was 9 months (SE = 0.574, 95% CI: 7.876–10.124), and that of the high-expression group was 8 months (SE = 0.912, 95% CI: 6.213–9.787).

**Fig. 4:**
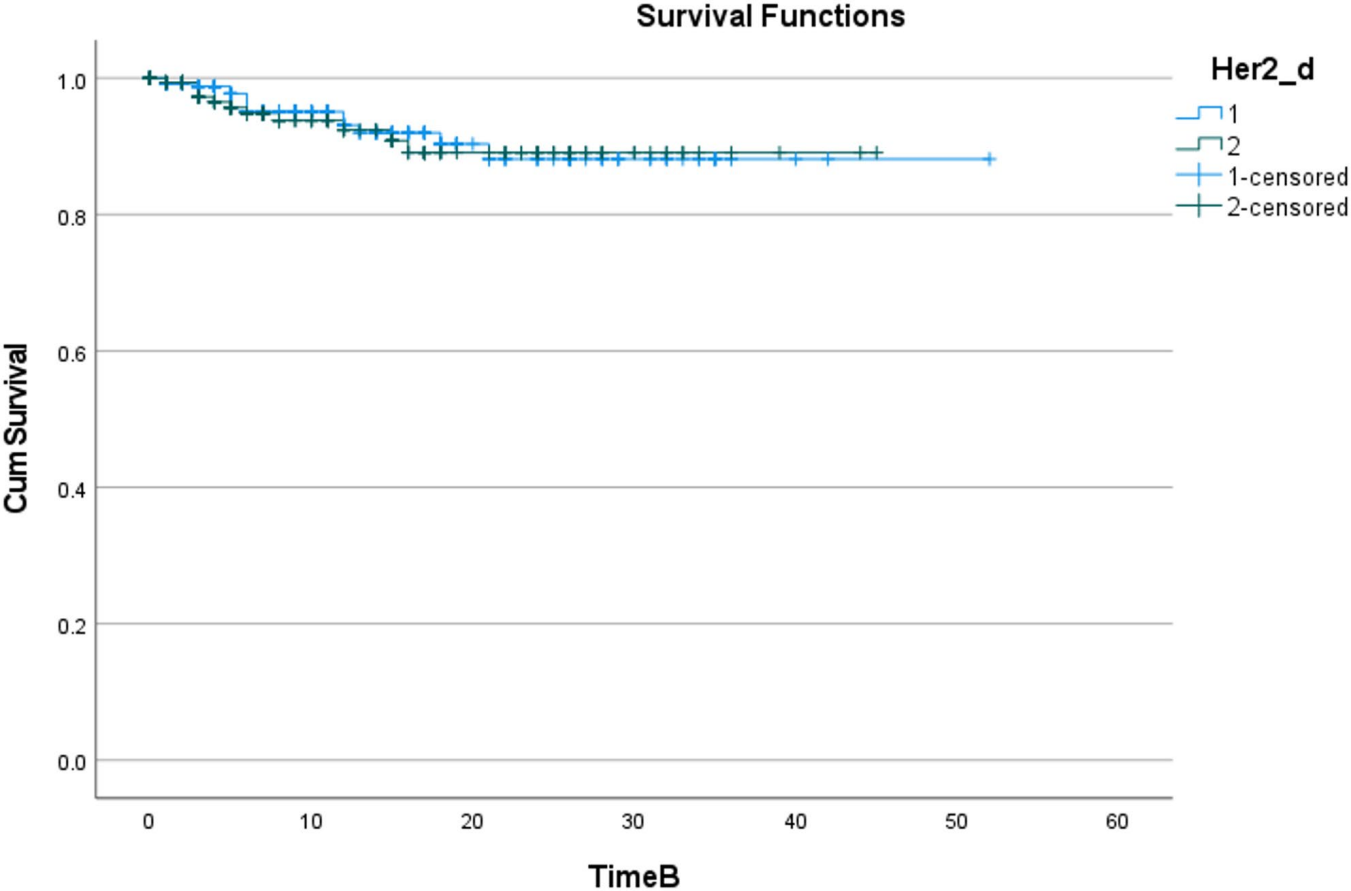
Metastasis Survival Curves of Patients with Different HER2 Expression Statuses

The Kaplan-Meier method was used to draw the cumulative survival curve of patients in different HER2 groups (as shown in Fig. 4), and the Log-Rank test was used to compare the survival difference between groups. The results showed that there was no statistical difference in the cumulative survival distribution between patients in different HER2 groups (χ²=0.065, df = 1, *P* = 0.799); the shape of the survival curve also suggested that the two curves almost overlapped, and different levels of Her2_d had no significant distinguishing effect on survival outcomes.

**Fig. 5:**
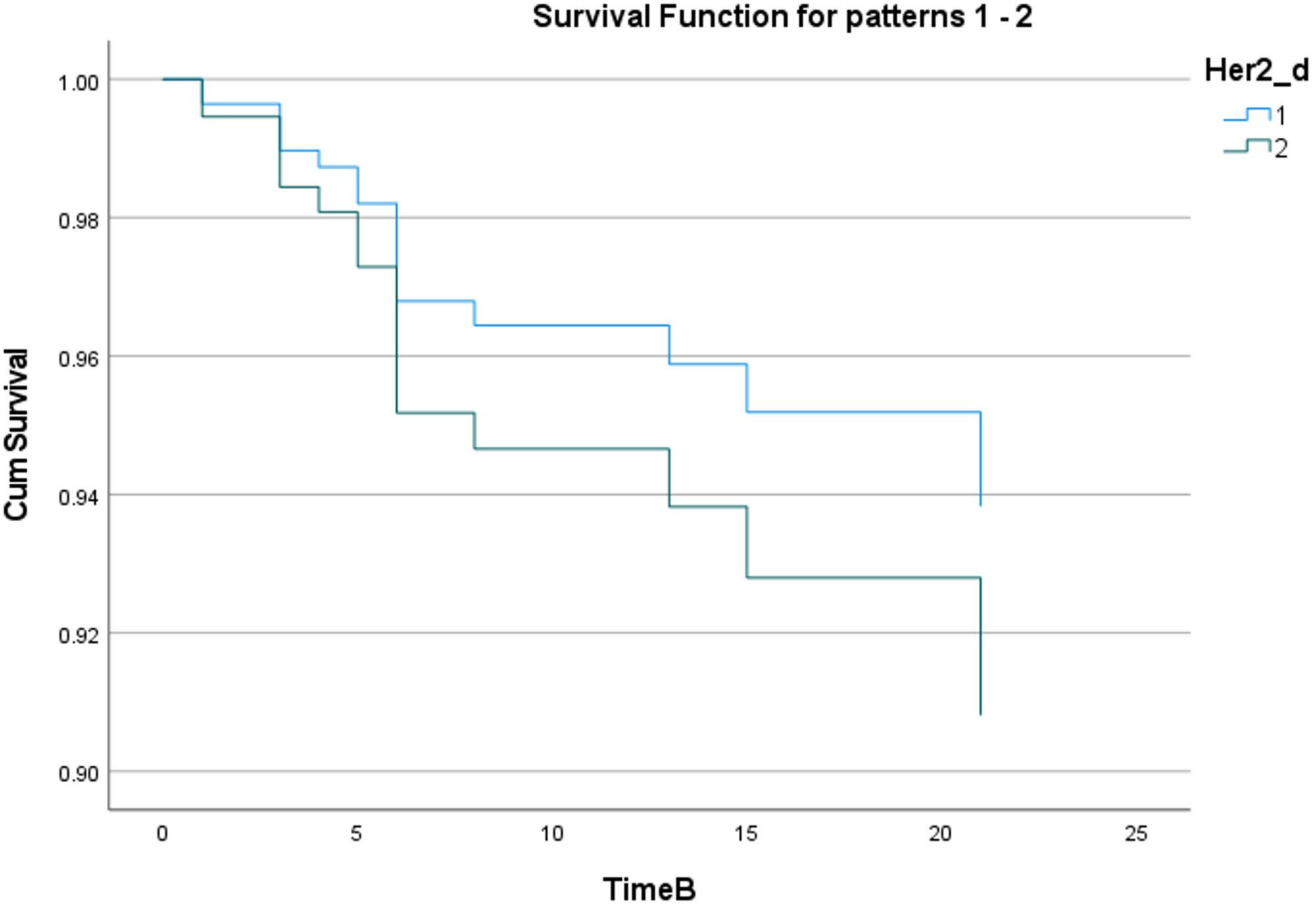
Metastasis Cox Proportional Hazards Regression Models of Patients with Different HER2 Expression Statuses

A multivariate Cox proportional hazards regression model was further constructed, including Her2_d, Age, Gender, and a number of hematological indicators (white blood cell count, neutrophil count, hemoglobin, etc.), to analyze the independent impact of each factor on outcome risk (as shown in Fig. 5). The Omnibus test of model coefficients showed that after including the variables, the combined predictive effect of the model on outcome risk was not statistically significant (overall chi-square = 18.033, df = 14, *P* = 0.205), suggesting that the overall explanatory power of the model on outcome risk was limited. After adjusting for other factors, there was no significant correlation between Her2_d and outcome risk (Wald χ²=0.665, df = 1, *P* = 0.415; HR = 1.515), that is, different levels of Her2_d did not show an independent impact on outcome risk.

## Discussions

This study, based on a retrospective analysis of 444 patients with urothelial carcinoma(UC) from a single center in Fujian Province, systematically evaluated the association between human epidermal growth factor receptor 2(HER2) expression status and clinicopathological characteristics and prognosis, providing a clinical basis for precise diagnosis and treatment of regional urothelial carcinoma.

HER2 is a member of the human epidermal growth factor receptor family, a transmembrane receptor protein with tyrosine kinase activity. It receives external growth signals on the cell surface and regulates cell growth, differentiation, and survival by activating intracellular signaling pathways such as PI3K. When HER2 gene amplification or protein overexpression occurs, it leads to abnormal cell signaling, thereby promoting tumor initiation and progression [9].

The results showed that the proportion of high HER2 expression (IHC 2+/3+) in this study population was 40.05%, which differs significantly from studies in different regions and populations. Bellmunt et al. (2015) studied two cohorts in Spain and Greece and found that the HER2 IHC 3 + positive rates were 22% and 4%, respectively, and the FISH amplification rates were 20% and 4%, clearly confirming significant geographical heterogeneity in HER2 expression [10]. Similar differences exist in studies from different provinces in China: Zhu et al.(2024) reported that the HER2 positivity rate (IHC 2+/3+) in high-grade urothelial carcinoma patients in Shandong Province reached 68.7% [11]; Zhou et al.(2023) found that the HER2 positivity rate in urothelial carcinoma in Beijing was 44%, and it was significantly higher in bladder cancer patients(51%) than in upper tract urothelial carcinoma patients(38%) [12]. These differences may be related to detection standards, sample baseline characteristics (such as tumor location and grade), and regional population genetic backgrounds.

Among the baseline clinical characteristics of patients, Age, History of bladder cancer, and Routine Urine Test were significantly associated with high HER2 expression in urothelial carcinoma patients. A weak but statistically significant positive correlation was observed between HER2 expression and age (Spearman’s ρ = 0.125, *p* = 0.009), suggesting that older patients are more likely to exhibit HER2 overexpression. Currently, there are few studies on the “association between HER2 and Age” in the field of urothelial carcinoma. Although this result is a weak association, it is statistically significant(*p* < 0.01), not only supplementing the clinical associated factors of HER2 expression in urothelial carcinoma but also providing a reference for precise diagnosis and treatment of patients of different ages. Additionally, the higher the values of Urine White Blood Cells (per HP), Urine Red Blood Cells (per HP), and Urine Tubular Epithelial Cells (per HP) in the patient’s Routine Urine Test, the greater the likelihood of high HER2 expression.

In pathological data, Pathological Grade, Ki67, and p53 were significantly associated with high HER2 expression in urothelial carcinoma patients. The Spearman’s rank correlation coefficient between HER2 and Pathological Grade was 0.434(*p* = 0.000), indicating a relatively strong association, suggesting that patients with high Pathological Grade(high-grade) are more likely to have high HER2 expression. This is consistent with clinical perception—high-grade urothelial carcinoma has stronger invasiveness. The Spearman’s correlation coefficients between HER2 expression and Ki67 and p53 were 0.372(*p* = 0.000) and 0.145(*p* = 0.015), respectively, suggesting that the higher the expression of Ki67 and p53, the greater the likelihood of high HER2 expression. Ki67 is a core marker of cell proliferation; the positive correlation between the two indicates that tumors with high HER2 expression are often accompanied by more active cell proliferation, further validating the driving role of HER2 in urothelial carcinoma cell proliferation. And p53 abnormality may indirectly promote HER2 gene amplification or upregulation by increasing genomic instability. From a molecular mechanism perspective, Moasser(2007) confirmed that HER2 can form heterodimers with HER3, activating downstream signaling pathways such as PI3K/Akt and Ras/MAPK, promoting cell proliferation, inhibiting apoptosis, and ultimately driving malignant tumor progression [9], which provides mechanistic support for the clinical phenomenon that “high HER2 expression is associated with high Pathological Grade and elevated Ki67(a cell proliferation marker).”

Meanwhile, this study found a correlation between HER2 expression and the choice of postoperative treatment regimens. Analysis showed that the Spearman’s correlation coefficients between HER2 expression and Immediate Perfusion after Surgery, Postoperative Perfusion Modality, and Neoadjuvant Chemotherapy modality were 0.126(*p* = 0.008), 0.22(*p* = 0.002), and 0.115(*p* = 0.035), respectively, suggesting a certain correlation between the degree of HER2 expression and the selection of treatment plans. This is consistent with the conclusion of Bellmunt et al. (2015)—their study indicated that HER2 status may influence clinical treatment decisions, especially in the formulation of postoperative adjuvant therapy regimens [10]. Additionally, Zhu et al. (2024) found that HER2 positivity was significantly associated with higher clinical stage in urothelial carcinoma(*p* = 0.019) [11], further corroborating the association between HER2 and disease malignancy in this study, suggesting that HER2 may serve as a potential marker for assessing the biological behavior of urothelial carcinoma.

In terms of prognosis analysis, this study confirmed through a multivariate Cox regression model that high HER2 expression is an independent risk factor for postoperative Recurrence in urothelial carcinoma patients (HR = 2.496, *P* = 0.044). After controlling for Age, Gender, and other hematological indicators, Her2_d was an independent risk factor for Recurrence in urothelial carcinoma (Wald χ²=4.058, df = 1, *P* = 0.044; HR = 2.496), meaning that when the Her2_d group was high expression, the Recurrence risk of patients was 2.496 times that of the low expression group. High HER2 expression as an independent risk factor for Recurrence implies that such patients are priority candidates for postoperative adjuvant targeted therapy. Zhou et al. (2023) also mentioned in their study on urothelial carcinoma in Beijing that HER2 expression is associated with patient Recurrence risk, and targeted therapy can reduce the Recurrence rate in high-expression patients [12]. This result differs from the conclusion of Bellmunt et al.(2015)’s multicenter study, which found no significant association between HER2 positivity and overall survival(OS) in both the Spanish(*P* = 0.49) and Greek(*P* = 0.12) cohorts [10], which may be related to the small sample size of previous studies or differences between regions and races. It is worth noting that Zhou et al. (2023) proposed that with the clinical application of HER2-targeted drugs(such as disitamab vedotin), HER2 expression is no longer a poor prognostic factor for urothelial carcinoma; instead, prognosis may improve due to patients benefiting from targeted therapy [12]. This study controlled for confounding factors such as Age, Gender, and multiple hematological indicators in the Recurrence analysis, and the results have certain robustness.

Regarding the relationship between HER2 and Metastasis in urothelial carcinoma, after controlling for other factors, Her2_d showed no significant association with Metastasis risk (Wald χ²=0.665, df = 1, *P* = 0.415; HR = 1.515), meaning that different levels of Her2_d did not show an independent effect on Metastasis risk. This result may be related to the small number of Metastasis cases or to some patients receiving subsequent targeted therapy or other individualized interventions, but its independent impact on long-term survival still requires further validation through studies with larger sample sizes and longer follow-up periods.

Compared with previous studies, the advantage of this study lies in the relatively large sample size(*n* = 444) and the inclusion of complete clinicopathological and follow-up data(such as Routine Urine Test, Pathological Grade, treatment plans, etc.), enabling a relatively systematic evaluation of the clinical significance of HER2 expression. However, this study still has certain limitations: first, as a single-center study, the generalizability of its results may be limited—Bellmunt et al.(2015)’s multicenter study, even with uniform detection methods, still found geographical differences in HER2 expression [10], and future verification through multicenter, prospective studies is needed; second, this study did not perform centralized uniform testing for HER2 expression, nor did it conduct further subgroup analysis of HER2-positive patients based on the results of IHC and FISH tests, which may lead to detection bias. Subgroups with different combinations of test results may differ in prognostic prediction and sensitivity to targeted therapy.Zhou et al.(2023) also found low consistency between HER2 IHC and FISH in urothelial carcinoma(only 16.7% of IHC 2 + patients were FISH positive) [12], highlighting the importance of a unified testing platform and standardized interpretation processes; furthermore, existing studies have confirmed that upper tract urothelial carcinoma and bladder cancer exhibit significant differences in epidemiological characteristics, clinicopathological manifestations, and prognosis, and there is a clear distinction in their HER2 positivity rates [12],. The lack of site-specific stratification may mask the heterogeneity in the role of HER2 in tumors at different sites; fourthly, patients were not divided into subgroups of superficial urothelial carcinoma and muscle-invasive urothelial carcinoma based on tumor invasion depth. These two subtypes differ significantly in biological behaviors, treatment strategies, and prognosis [2], and the driving role of HER2 in tumors with different invasion depths may vary; fifthly, the lack of HER2 status in metastatic lesions limits in-depth exploration of tumor heterogeneity, and it is unclear whether HER2 expression is consistent between primary and metastatic lesions, which is also a direction for future research improvement.

In summary, HER2 expression is relatively common in urothelial carcinoma patients in Fujian Province (40.05%), and it is closely related to high malignant phenotypes such as Age, History of bladder cancer, and Pathological Grade, especially as an independent risk factor for increased Recurrence risk, suggesting that it may serve as an important reference indicator for postoperative management. Future research should further explore the molecular mechanisms of HER2 in urothelial carcinoma (such as its interaction with the PI3K/Akt pathway) and its potential value in targeted therapy, particularly the application prospects of antibody-drug conjugate (ADC) drugs in HER2-low expression patients. Meanwhile, establishing a unified HER2 testing platform and standardized interpretation processes will help promote the development of individualized treatment for urothelial carcinoma.

## Conclusions

By analyzing data from 444 patients with urothelial carcinoma using multiple methods, this study found that high HER2 expression was positively correlated with clinicopathological characteristics such as age and history of bladder cancer, as well as indicators related to malignant progression; HER2 expression level was associated with clinical treatment decisions including postoperative perfusion and neoadjuvant chemotherapy; multivariate analysis confirmed that high HER2 expression was an independent risk factor for recurrence of urothelial carcinoma but had no significant correlation with metastasis risk. These findings suggest that HER2 is more suitable as a potential marker for recurrence risk stratification in urothelial carcinoma patients rather than a definitive generalized prognostic factor. The study has limitations such as a limited sample size and failure to fully integrate MRI imaging features and HER2 isoform subtypes, which may affect the generalizability and accuracy of the conclusions. In the future, the sample size can be expanded to construct a comprehensive risk stratification model combining HER2 and imaging features, conduct efficacy studies on HER2-targeted therapy and synergistic immunotherapy in patients with different risk stratifications, explore the impacts of HER2 isoform subtypes and spatial expression heterogeneity, and combine dynamic monitoring of circulating tumor DNA to promote the translation of research results into clinical precise diagnosis and treatment.

## Supplementary Information

Supplementary Material 1.

## Data Availability

The datasets generated for this study are available to the corresponding author upon request.

## Abbreviations

HER2: Human epidermal growth factor receptor 2
UC: Urothelial carcinoma
TURBT: Transurethral resection of bladder tumor
RC: Radical cystectomy
OS: Overall survival
PFS: Progression-free survival
IHC: Immunohistochemistry
HR: Hazard ratio
CI: Confidence interval
