# Supplementary figures and images for: Correlation analysis of HER2 expression with clinicopathological features and prognosis based on data from 444 patients with urothelial carcinoma
Source: BMC Urol. 2026 Feb 24;26:86. doi: 10.1186/s12894-026-02088-3 (PMC13036885; doi:10.1186/s12894-026-02088-3)

Supplementary Figure X


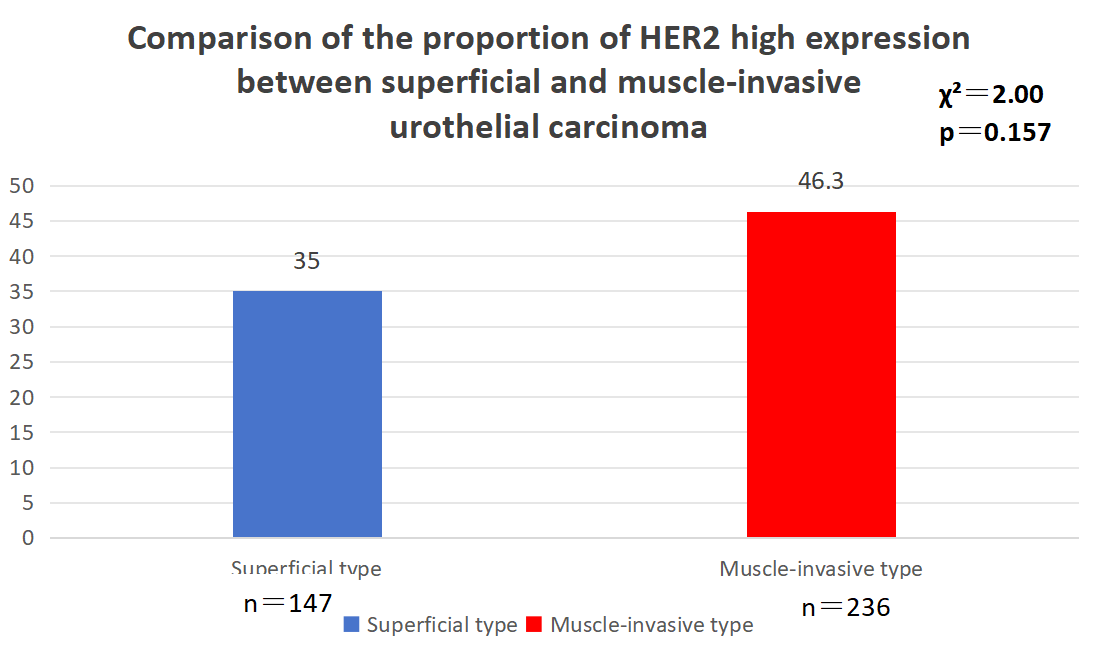


Supplementary Figure Y


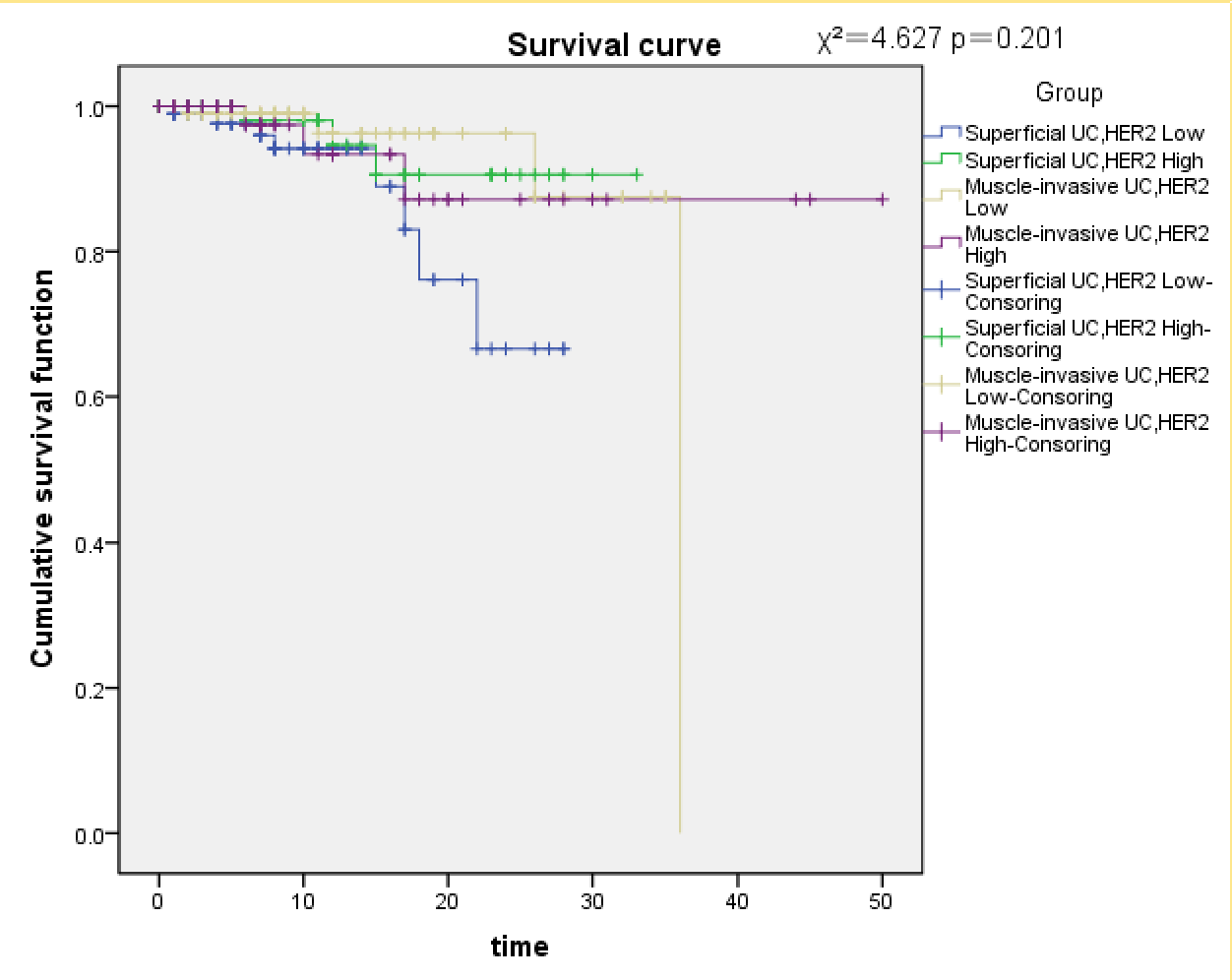

Supplement: Supplementary file 1 — Supplementary Material 1. [file 12894_2026_2088_MOESM1_ESM.docx]
